# 

**Published:** 1889-09-28

**Authors:** 


SATURDAY, SEPTEMBER 28th, 1889.

**Dr. Nansen's Message** - 399

**Words of Consolation**—Sickness - 400

**The Doctor's Pulpit.**—Fun at Fifty - 401

**The Story of the Insane from Year to Year.**—Asylum Reports - 402

**Women Physicians at Clapham** - 403

**Within the Wards.**—About Specialists and Specialism - 404

**The Deaf and the Dumb** - 405

**Notes and News** - 406

**Everybody's Page** - 408

**Annotations.**—All the World over—Hypnotism and Theosophy—The Luxury of Fresh Eggs - 409

**Her Heart's Mistake.** By E. D. CUMMING. Conclusion - 410

**The Amines Treatment of Sewage** - 413

**Amusements and Relaxation** - 414

**Scraps and Gleanings** - 414

**EXTRA SUPPLEMENT—THE NURSING MIRROR.**

**En Passant.**—News from the East—Marriage of a Matron—The Doll Competition—Attendants on the Insane—Short Items—Puerperal Fever - xcix
**Case Taking.**—First Paper - c
**A Probationer's Diary** - c
**Doll Competition** - ci
**Everybody's Opinion.**—Combination v. Insurance Nursing the Insane—Superannuated at Forty-Three—Norfolk and Norwich Hospital—Nurses' Trials—Asylum Attendant - ci
**Appointments—Vacancies—Notes and Queries** - cii

